# Primary care consultations and pain medicine prescriptions: a comparison between patients with and without chronic pain after total knee replacement

**DOI:** 10.1186/s12891-022-05492-6

**Published:** 2022-06-07

**Authors:** Rafael Pinedo-Villanueva, Spyros Kolovos, Christopher Maronga, Antonella Delmestri, Nick Howells, Andrew Judge, Rachael Gooberman-Hill, Vikki Wylde

**Affiliations:** 1 Nuffield Department of Orthopaedics, Rheumatology and Musculoskeletal Sciences, Nuffield Orthopaedic Centre, University of Oxford, Windmill Road, OX3 7LD Oxford, UK; 2 Orthopaedic Department, North Bristol NHS Trust, Bristol, UK; 3 Musculoskeletal Research Unit, Bristol Medical School, University of Bristol, Bristol, UK; 4 Bristol Biomedical Research Centre, University Hospitals Bristol and Weston NHS Foundation Trust and the University of Bristol, Bristol, UK

**Keywords:** Total knee replacement, Chronic pain, Primary care, Opioid, England

## Abstract

**Background:**

Approximately 20% of patients experience chronic pain after total knee replacement (TKR). The impact of chronic pain after TKR on primary care services in the UK is currently unknown. The aim of this study was to compare primary care consultations and pain medicine prescriptions between patients with and without chronic pain after TKR.

**Methods:**

Data from 5,055 patients who received TKR between 2009 and 2016 with anonymised linked data from the Clinical Practice Research Datalink Gold (CPRD) and English Hospital Episode Statistics (HES) Patient Reported Outcome Measures (PROMs) programme were analysed. The exposure time was from 10 years pre-operative to eight years post-operative. Patients with a score ≤ 14 on the Oxford Knee Score pain component scale at 6 months post-operative were classified as having chronic pain after TKR. Primary care consultations and prescribed pain medicines were quantified, and costs calculated based on national cost data.

**Results:**

721 patients (14%) had chronic pain after TKR. The prevalence and costs of primary care consultations and pain medicine prescriptions per year were consistently higher for patients with chronic pain after TKR compared with those without chronic pain after TKR; these differences were observed both before and after surgery. There was a substantial and sustained increase in the cost of opioid prescriptions after surgery for patients with chronic pain after TKR, peaking at seven years post-operative.

**Conclusions:**

Increased primary care consultations and pain medicine prescriptions associated with chronic pain after TKR represent a considerable financial cost to primary care services. Evaluation of interventions to reduce the risk of developing this pain condition and improve the early management of pain after TKR are needed to improve outcomes for patients and reduce costs to healthcare services.

**Supplementary Information:**

The online version contains supplementary material available at 10.1186/s12891-022-05492-6.

## Background

Osteoarthritis is the most common musculoskeletal condition worldwide and is the leading cause of disability in the UK, with one third of people aged ≥ 45 year old having sought treatment for their osteoarthritis [1]. It is also associated with substantial healthcare and societal costs [2]. The National Institute for Health and Care Excellence (NICE) Clinical Guideline for osteoarthritis [3] recommends patients are provided with information and individualised self-management strategies are discussed. Core treatments include exercise, weight loss (if appropriate), use of appropriate aids and devices, pain medications (paracetamol and non-steroidal anti-inflammatory drugs as first line treatment for pain) and intra-articular corticosteroid injections. If these treatments are not effective at controlling pain, then patients can be referred to secondary care for consideration for joint replacement.

TKR is one of the most common elective operations; it has been estimated that nearly five million people were living with a TKR in the USA in 2010 [4]. In the UK National Health Service (NHS), over 100,000 operations are performed annually [5, 6]. Due to changes in population demographics and obesity, the rate of TKR has been predicted to increase [7]. The principal aim of surgery is to improve function and provide relief from chronic knee pain [5]. For many patients, TKR is successful at providing pain relief, however approximately 20% of patients experience chronic post-surgical pain, defined as pain that occurs or increases in intensity at three months or longer after surgery [8]. Chronic pain after TKR is associated with reduced quality of life and functional limitations [9], and people are often disappointed with their outcome, struggle to make sense of their pain and may not seek help [10, 11].

In the UK, management of chronic pain after TKR is provided within primary and secondary care services. Primary care services provide the first point of contact in the healthcare system and are free at the point of delivery and receipt. Research indicates that 32% of healthcare costs in the second year after TKR are primary care costs [12]. However, the impact of chronic pain after TKR on primary care services has not yet been explored. The aim of this study was to compare primary care consultations and prescribed pain medicines between patients with and without chronic pain after TKR by analysing routine NHS healthcare data.

## Methods

### Study design

This was a retrospective observational study using anonymised linked data from the Clinical Practice Research Datalink Gold (CPRD), English Hospital Episode Statistics (HES) Patient Reported Outcome Measures programme (PROMs), HES Admitted Patient Care, HES outpatient, Office for National Statistics (ONS) mortality data and Index of Multiple Deprivation 2015. All methods were performed in accordance with the relevant guidelines and regulations and reporting of the findings adhere to the STrengthening the Reporting of OBservational studies in Epidemiology (STROBE) guidelines.

### Data sources

The CPRD dataset contained anonymised information on > 14 million patients registered at 714 UK general practices. Data include computerised records of all consultation and prescription events in primary care and comprehensive demographic information. CPRD provides access to HES-PROMs data held under the CPRD Data Linkage Scheme. HES holds information on patients admitted to NHS hospitals in England, including diagnostic International Classification of Diseases (ICD) codes providing information about a patient’s condition and OPCS Classification of Interventions and Procedures version 4 procedural codes for surgery. Since April 2009, HES provides PROMs data on patients undergoing knee replacement in English NHS hospitals, including a preoperative and 6 month post-operative quality of life questionnaire (EQ-5D) and joint-specific PROM (Oxford Knee Score; OKS).

### Sample

Our linked CPRD-HES-PROMs dataset included all NHS patients with a CPRD record for a primary TKR between 2009 (when PROMs data collection began) and 2016 (when the data were extracted). Analysis was limited to patients with linked HES records who completed a 6-month post-operative OKS. A data science tool was used to perform automated data engineering, data mining and advanced curation on CPRD and HES data cuts. The tool received raw data as input and provided as output selected and structured information ready for analysis.

### Definition of chronic pain after TKR

The OKS is a validated 12-item joint-specific questionnaire that assesses knee pain and function in patients undergoing TKR [13]. A 7-item OKS pain component score (OKS-PS) [14] can be derived from the OKS, with scores ranging from 0 to 28 (worst to best). We have previous derived a cut-off point on the OKS-PS to identify patients with chronic pain after TKR that can be used for patient selection in a research setting. Using data from the English PROMs programme, we found that individuals with a score of ≤ 14 on the OKS-PS have pain that is likely to negatively impact on their health-related quality of life [15]. These patients had pain that was characterised by frequent and severe problems in all pain dimensions on the OKS, particularly pain severity, night pain and limping, and in all dimensions of health-related quality of life. This cut-off point has subsequently been used in a multi-centre randomised controlled trial to identify patients with pain at three months after TKR who would likely benefit from intervention [16]. For the current analyses, we applied the cut-off point to classified patients based on their 6-month post-operative OKS-PS as either having chronic pain after TKR (score of 0–14) or not having chronic pain after TKR (score of 15–28).

### Primary care consultations

A primary care consultation was defined as any direct health-related encounter with a primary care healthcare professional. Given the large number of staff roles (Additional file), these were grouped into General Practitioners (GPs), nurses, and other. Consultation types classed as direct health-related encounters were included (detailed criteria in the Additional file). Reason for consultation is not recorded in CPRD, therefore all direct health-related consultations were included in the analyses. Costs for the primary care consultations were calculated based on mean national unit cost of consultation with respective healthcare staff as reported in the Personal Social Services Research Unit [17].

### Prescribed pain medicines

Included medicines were those identified using a list developed with expert clinicians based on their views about which medications would be prescribed to patients with pain related to their knee. Pain medicines were grouped into paracetamol (acetaminophen), antidepressants, non-steroidal anti-inflammatories (NSAIDs), and opioids (Additional file). To calculate costs, we searched the British National Formulary website for each medication (e.g. https://bnf.nice.org.uk/medicinal-forms/paracetamol.html) and extracted unit cost based on strength and number of units. Based on the unit cost and quantity prescribed as reported in CPRD records, the total cost of each pain medicine prescription was calculated.

### Analyses

Exposure time was defined as the period valid CPRD data were collected for patients before and after TKR; this varied from patient to patient because of different start and end dates linked to surgery data collection; quality of data; and patient registration, change, or death. Death was calculated using CPRD GOLD and ONS linked data using a published algorithm [18]. Reporting is by year before and after surgery as long as the patient was active during that 12-month period. Sensitivity analyses were conducted adjusting for exposure time, such that values over partial years were inflated as an estimate of a full-year resource use or cost. Analyses were conducted over the period between 10 years before and eight years after TKR; after that the sample of active patients dropped below 500 and the number of patients with chronic pain was deemed too low (Additional file).

Descriptive analyses were undertaken to report patients’ demographic and clinical characteristics. Primary care consultations and pain medicine prescriptions were calculated for patients with and without chronic pain after TKR. Resource use was calculated as the mean number of primary care consultations per patient per year for any cause and associated costs were calculated as the mean cost per patient per year. Pain medicine prescription costs were calculated as mean cost per patient per year. Some specific medicinal forms were excluded because formulation was not costed or because the quantity was not available. In all such cases, the number of prescriptions excluded as a percentage of the total is reported. To assess whether costs between chronic pain groups were different, bootstrap confidence intervals were calculated for over 1,000 samples with replacement for yearly consultation and prescription costs. Bootstrapped methods were used because cost data are right-skewed and standard parametric methods are hence not appropriate. Differences in outcomes between men and women were assessed via generalised estimating equations (GEE) models using the Gamma distribution and log link function. Analyses were conducted using R.

## Results

### Patients

A total of 5,055 patients were included in the analyses. Of these, 721 (14.3%) had a score of ≤ 14 on their 6-month post-operative OKS-PS and were classified as having chronic pain after TKR. Patient demographics and clinical characteristics are displayed in Table 1. Patients had a mean age at surgery of 69 (standard deviation 9) and 56% were female. Mean BMI was 31 (standard deviation 5), 6% of patients were current smokers, 91% had a Charlson Comorbidity score of 0 in the past year and 10% were in the lowest quintile of the IMD deprivation score. The mean exposure time was four years (standard deviation 3). Differences in characteristics between the chronic pain and non-chronic pain group included: younger age, higher BMI, higher frequency of smokers and non-drinkers, two or more co-morbidities in the past five years, and live in areas of higher deprivation area (Table 1).

**Table 1:** Demographic and clinical characteristics of the sample population

	Total (*n* = 5,055)	Chronic pain patients (*n* = 721)	Non-chronic pain patients (*n* = 4334)	*P*-values for significance tests
Age: mean (SD)	69 (9)	67 (10)	69 (9)	< 0.001^a^
Female: n (%)	2849 (56%)	420 (58%)	2429 (56%)	0.286^b^
BMI: mean (SD)	31 (5)	32 (6)	30 (5)	< 0.001^a^
Exposure time (years)	4 (3)	4 (3)	4 (3)	0.003^c^
Current smoker: n (%)	302 (6%)	97 (14%)	205 (5%)	< 0.001^b^
Current drinker: n (%)	2528 (79%)	331 (72%)	2197 (80%)	< 0.001^b^
Charlson Comorbidity: n (%) (5 years prior)				
None	3570 (71%)	472 (65%)	3098 (71%)	< 0.001^b^
1	423 (8%)	61 (8%)	362 (8%)	
2	614 (12%)	99 (14%)	515 (12%)	
3+	448 (9%)	89 (12%)	359 (8%)	
Charlson Comorbidity: n (%) (1 year prior)				
None	4619 (91%)	644 (89%)	3975 (92%)	0.096^b^
1	209 (4%)	34 (5%)	175 (4%)	
2	186 (4%)	33 (5%)	153 (4%)	
3+	41 (1%)	10 (1%)	31 (1%)	
IMD deprivation score: n (%) (quintiles; least to most deprived)				
1	1233 (24%)	119 (17%)	1114 (26%)	< 0.001^b^
2	1313 (26%)	162 (22%)	1151 (27%)	
3	1136 (22%)	168 (23%)	968 (22%)	
4	844 (17%)	140 (19%)	704 (16%)	
5	523 (10%)	131 (18%)	392 (9%)	

^Missing data: BMI = 898 missing; current smoker = 413 missing; current drinker = 1882 missing; IMD deprivation score = 6 missing^

^a^ t-test, ^b^ Chi-square test, ^c^ Wilcox test

### Primary care consultations

**Fig. 1:**
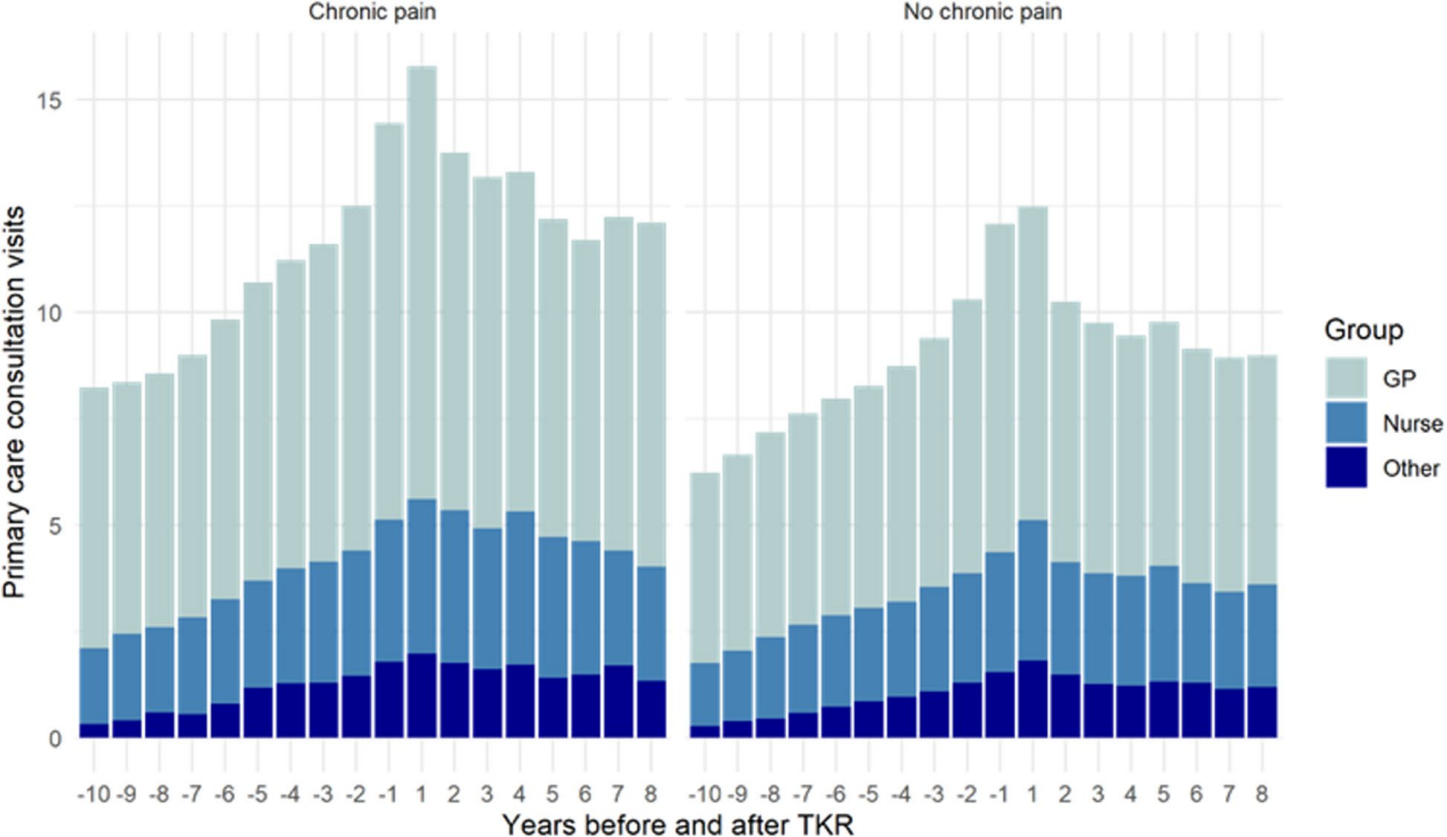
Mean number of consultations by healthcare professional for patients with and without chronic pain

The mean number of primary care consultations for patients with and without chronic pain after TKR from the 10 years before surgery to eight years after surgery is displayed in Fig. 1. The mean number of primary care consultations per patient was highest in the year immediately before and immediately after TKR; this pattern was observed for patients with and without chronic pain after TKR. The mean number of primary care consultations per year was consistently higher in patients with chronic pain compared to those without chronic pain after TKR; this pattern was consistent across consultations with different types of healthcare professionals (Additional file).

**Fig. 2:**
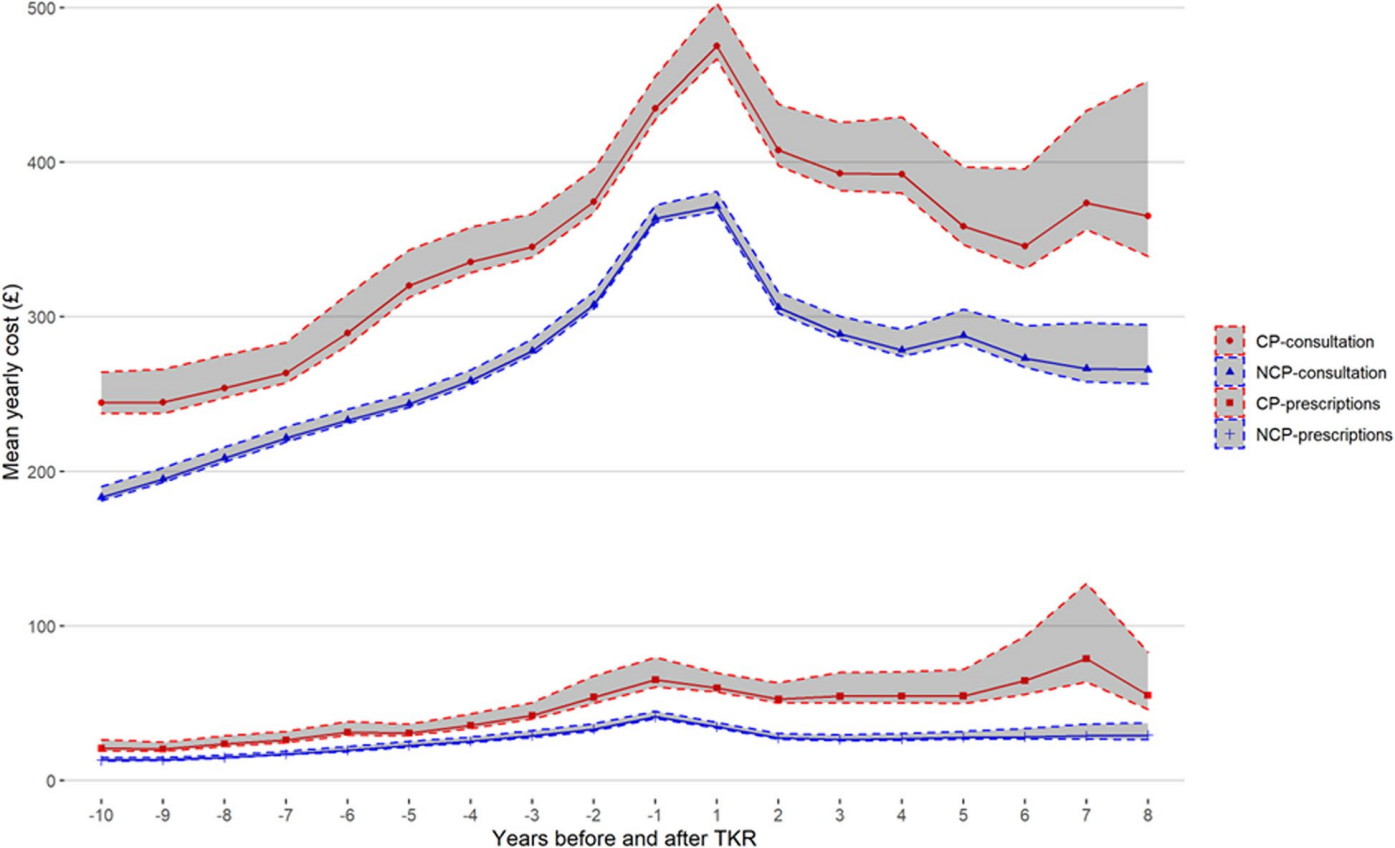
Mean cost of consultations and prescriptions by year for patients with and without chronic pain

The mean primary care consultation costs per year for patients with and without chronic pain after TKR, overall and by healthcare professional category, are provided in Table 2. Primary care consultation costs were significantly different between groups throughout the 18 years of analysis, as shown by the non-overlapping bootstrap confidence intervals shown in Fig. 2. In the 10 years prior to TKR, the mean cost per year in primary care consultations for patients who would go on to develop chronic pain after TKR was £42-£77 higher than the mean yearly cost of patients who did not develop chronic pain after TKR. Post-operatively, this excess mean yearly cost between patients with and without chronic pain was even higher, ranging from £71 to £114. Mean consultations costs per patient increased between the year prior and the year immediately following surgery. The change was significant for the chronic pain group (mean £435, CI £427-£456 during the year prior and mean £475, CI £467-£503 the year after TKR) but not for those without chronic pain (see Table 2). The main driver of the cost differences in primary care consultations between patients with and without chronic pain were the higher use of GP appointments by patients with chronic pain. The GEE model adjusting for age and year showed that the cost of consultations for men was significantly lower (*p* < 0.001) than that of women. The difference persisted after controlling for chronic pain group (*p* < 0.001) and drinking and smoking (*p* < 0.001). A figure showing sex-stratified mean yearly consultation costs with bootstrap confidence intervals by chronic pain group is shown in the Additional file.

**Table 2:** Mean costs of primary care consultations by year for patients with and without chronic pain

	With chronic pain				Without chronic pain			
**Year**	**GPs**	**Nurses**	**Other**	**Total (CI**^**a**^**)**	**GPs**	**Nurses**	**Other**	**Total (CI**^**a**^**)**
-10	£202	£29	£13	£244 (£237-£264)	£148	£24	£12	£183 (£181-£190)
-9	£195	£33	£16	£245 (£237-£266)	£152	£27	£16	£195 (£193-£202)
-8	£197	£32	£24	£254 (£248-£275)	£159	£31	£18	£209 (£206-£216)
-7	£204	£37	£23	£264 (£257-£283)	£164	£34	£24	£222 (£219-£229)
-6	£217	£40	£32	£289 (£281-£314)	£168	£35	£30	£233 (£231-£240)
-5	£231	£41	£48	£320 (£313-£343)	£172	£36	£35	£243 (£241-£251)
-4	£239	£44	£52	£335 (£328-£358)	£183	£37	£39	£258 (£256-£265)
-3	£246	£46	£53	£345 (£338-£367)	£193	£40	£45	£278 (£275-£285)
-2	£267	£48	£59	£374 (£367-£396)	£213	£42	£53	£307 (£305-£316)
-1	£308	£55	£72	£435 (£427-£456)	£255	£46	£63	£364 (£361-£372)
1	£336	£59	£80	£475 (£467-£503)	£243	£54	£74	£371 (£368-£381)
2	£277	£59	£72	£408 (£398-£438)	£202	£43	£60	£306 (£302-£316)
3	£273	£54	£66	£393 (£382-£426)	£195	£43	£52	£289 (£285-£300)
4	£263	£59	£70	£392 (£380-£429)	£186	£43	£50	£278 (£274-£292)
5	£247	£54	£58	£358 (£346-£397)	£189	£45	£54	£288 (£283-£305)
6	£234	£51	£61	£346 (£331-£396)	£182	£38	£53	£273 (£267-£294)
7	£259	£45	£70	£374 (£356-£434)	£182	£38	£47	£266 (£258-£296)
8	£267	£44	£55	£365 (£339-£453)	£177	£40	£49	£266 (£256-£295)

^a^ Bootstrap confidence interval

### Prescribed pain medicines

**Fig. 3:**
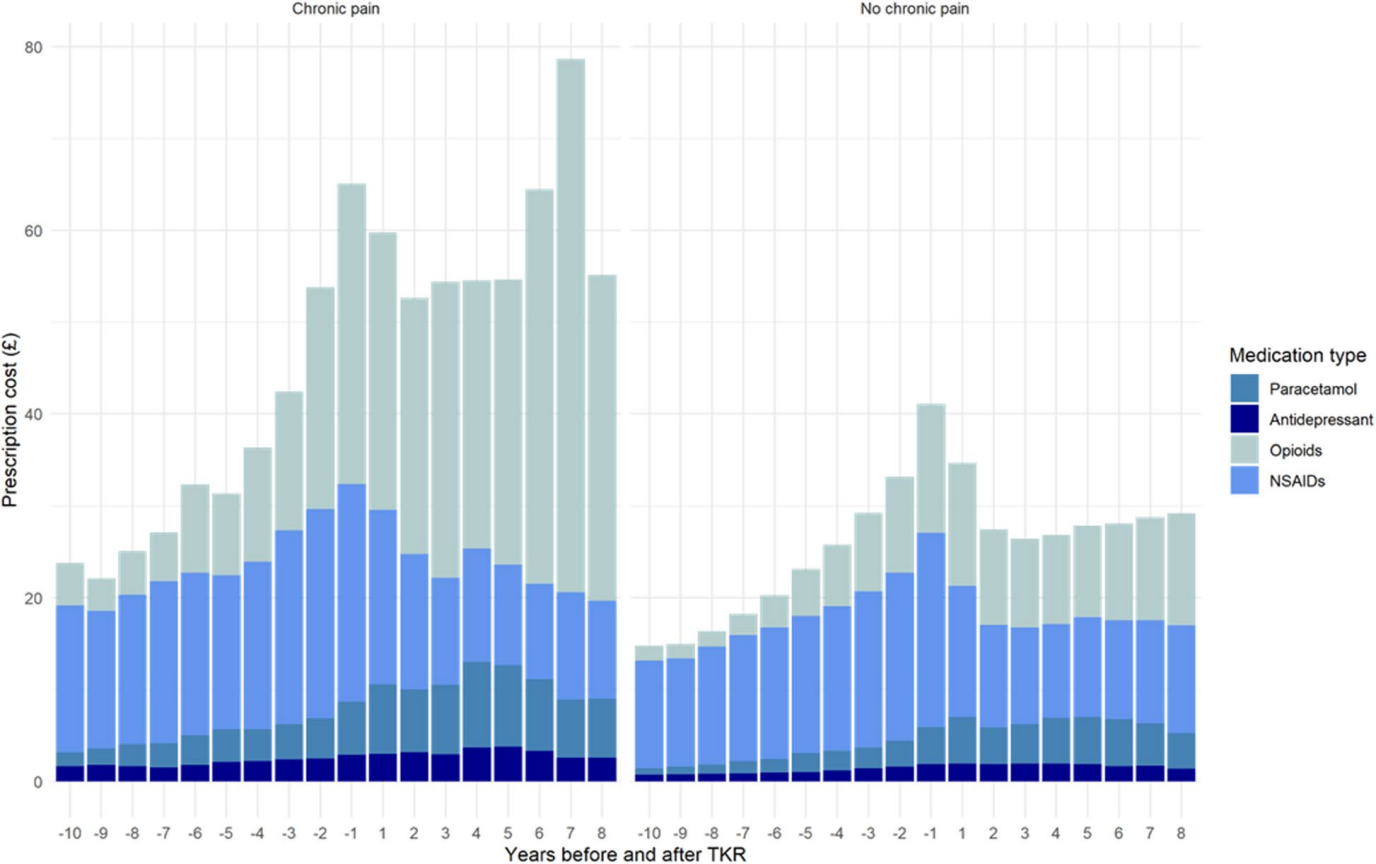
Mean cost of pain medicine prescriptions by year and medication type for patients with and without chronic pain

Yearly costs for paracetamol, antidepressants, NSAIDs and opioids for patients with and without chronic pain are summarised in Fig. 3 (further results are provided in the Additional file). Prescriptions costs for patients with chronic pain were significantly higher than those without chronic pain after TKR both before and after surgery, in fact for all 18 years of analysis (see Fig. 2). Stronger pain medication drove greater percentages of total prescription costs leading up to surgery. Mean pain prescription costs per patient dropped between the year prior and the year immediately following surgery. The change was not significant for the chronic pain group, but it was statistically significant for those without chronic pain (mean £41, CI £40-£45 during the year prior and mean £35, CI £34-£38 the year after TKR). Yearly confidence intervals for prescription costs by group are provided in the Additional file. Pain medicine prescription costs generally increased as patients reached surgery, and then they dropped only to increase again, slightly for patients without chronic pain but a larger increase for those with chronic pain after TKR. The GEE model adjusting for age and year alone showed that the cost of prescriptions for men was significantly lower (*p* = 0.018) than that of women. This was also the case after controlling for chronic pain group (*p* = 0.011) but the difference was not evident after adding drinking and smoking (*p* = 0.062). A figure showing the mean yearly prescription costs for men and women, with bootstrap confidence intervals by chronic pain group, is shown in the Additional file.

Of particular note, whilst the cost of opioid prescriptions decreased only slightly after TKR and remained relatively stable after that for patients without chronic pain, for those with chronic pain it increased, peaking at seven years post-operative. The proportion of patients receiving a prescription for opioids increased for both groups as they approached surgery (34% and 20% the year prior to TKR for patients with and without chronic pain, respectively), peaking the year after (52% and 32%). After that, the proportion of patients receiving a prescription for opioids averaged values similar to those observed the year before surgery.

## Discussion

Our analysis of English routine NHS healthcare data indicated that primary care consultations and prescribed pain medicines were consistently higher for patients with chronic pain after TKR compared to patients without chronic pain. These differences were evident during the entire observational period of our analyses, persisting from 10 years pre-operative until eight years post-operative. There was a considerable difference in the prescription of opioids in the period after surgery, with a steep increase in costs for patients with chronic pain after TKR which peaked at seven years post-operative.

It is well known that chronic pain is associated with high societal and healthcare costs, with the national cost of pain found to exceed the costs of heart disease, cancer and diabetes in the United States [19]. People living with chronic musculoskeletal pain make high use of healthcare and receive a large number of prescriptions for pain medicines [20, 21]. Our study is the first to specifically quantify the impact of chronic pain after TKR on English primary care services. Chronic pain after TKR poses a considerable cost to primary care, given the high prevalence and long-term nature of this kind of pain. The consistently higher rate of primary care consultations and pain medicine prescriptions in the 10-year pre-operative period by patients who developed chronic pain after TKR highlights an increased healthcare utilisation even before their surgery. It was not possible to determine causes for these differences within our study, however we observed that patients in the chronic pain group had a higher BMI and were more likely to be current smokers, have more co-morbidities and live in deprived areas. These are all factors that can contribute to poorer health and an associated increased use of health services, and previous research has also identified these as risk factors for the development of chronic pain after TKR [22, 23].

The cost of opioid prescriptions increased substantially after surgery for patients with chronic pain after TKR and remained high over the eight-year post-operative observational period. This suggests that opioids were prescribed with primary care for long-term management of chronic pain after TKR. However, the data analysed was from 2009 to 2016, and medical practice and prescribing is likely to have changed over time, reflecting the consensus amongst healthcare professionals that opioids are not indicated for chronic pain [24], with clear evidence that they provide minimal relief of chronic pain symptoms, including pain due to osteoarthritis, and are associated with considerable harm [25]. Therefore, although our data shows a recent historical trend of high opioid prescription costs within primary care for patients with chronic pain after TKR, further research is needed to evaluate current prescription patterns in light of recent national guidance advising against opioid prescription for patients living with chronic pain.

Our analyses used CPRD data, which contains detailed patient-level and prescription-level data on a large sample of patients from across England. However, limitations of our analyses should be acknowledged when interpreting the results. The data in CPRD are based on a subset of GP practices, and our analysis was restricted to those patients with linked HES records that completed a 6-month post-operative OKS, which may have introduced selection bias into our findings and limited generalisability. The data only contain GP prescriptions and not pharmacy dispensations, and therefore it was not possible to confirm the quantity of medications that were bought. We also did not capture the costs to patients of purchasing their own pain medication, and this is an area which may warrant further research. We defined chronic pain status at six months post-operative, a time point at which pain outcomes generally plateau after TKR [26]. However, we acknowledge that pain is rarely static and there can be within-person variability in longer-term outcomes [27] and we were unable to account for fluctuations in post-operative pain status over the 10 year follow-up period. Also, although we used a joint-specific and validated measure to assess knee-related pain, it was not possible to determine whether the knee pain reported by patients was directly related to their TKR or due to another cause.

In conclusion, our study demonstrated that increased primary care consultations and pain medicine prescriptions associated with chronic pain after TKR represent a considerable financial cost to primary care services in England and that patients with chronic pain after TKR are prescribed more opioid medications than those without chronic pain. Evaluation of interventions to reduce the risk of developing this pain condition and improve the early management of pain after TKR are needed to improve outcomes for patients and reduce costs to healthcare services.

## Supplementary Information

**Additional file 1.**

## Data Availability

The datasets analysed during the current study cannot be shared publicly because restrictions apply to the availability of these data, which were used under licence for the current study. CPRD Gold and HES-PROMS linked data are available through data applications to the Independent Scientific Advisory Committee for MHRA database research.

## Abbreviations

TKR: Total knee replacement
CPRD: Clinical Practice Research Datalink Gold
HES: Hospital Episode Statistics
PROMs: Patient Reported Outcome Measures
NICE: National Institute for Health and Care Excellence
NHS: National Health Service
ONS: Office for National Statistics
STROBE: STrengthening the Reporting of OBservational studies in Epidemiology
ICD: International Classification of Diseases
OKS-PS: Oxford Knee Score pain component score
GPs: General Practitioners
NSAIDs: non-steroidal anti-inflammatories
