# Supplementary material for: Primary care consultations and pain medicine prescriptions: a comparison between patients with and without chronic pain after total knee replacement
Source: BMC Musculoskelet Disord. 2022 Jun 7;23:548. doi: 10.1186/s12891-022-05492-6 (PMC9172077; doi:10.1186/s12891-022-05492-6)
Supplement: Supplementary file 1 — Additional file 1. [file 12891_2022_5492_MOESM1_ESM.docx]

**Supplementary materials**

Contents

[Table 1: Staff roles recording in CPRD 2](#_Toc99349753)

[Table 2: Inclusion/exclusion criteria for primary care consultations 3](#_Toc99349754)

[Specific drugs included in analysis 4](#_Toc99349755)

[Figure 1: Number of patients active within CPRD by year before and after primary TKR 5](#_Toc99349756)

[Figure 2: Mean number of primary care consultations by year for patients with and without chronic pain after TKR 6](#_Toc99349757)

[Figure 4: Mean number of nurse consultations by year for patients with and without chronic pain after TKR 7](#_Toc99349758)

[Figure 5: Mean number of other healthcare professional consultations by year for patients with and without chronic pain after TKR 7](#_Toc99349759)

[Table 3: Mean number of consultations for the different types of healthcare professionals by year for patients with and without chronic pain after TKR 8](#_Toc99349760)

[Figure 6: Paracetamol - Mean cost per patient per year for patients with and without chronic pain after TKR 9](#_Toc99349761)

[Figure 7: Antidepressants - Mean cost per patient per year for patients with and without chronic pain after TKR 9](#_Toc99349762)

[Figure 8: NSAIDs - Mean cost per patient per year for patients with and without chronic pain after TKR 10](#_Toc99349763)

[Figure 9: Opioids - Mean cost per patient per year for patients with and without chronic pain after TKR 10](#_Toc99349764)

[Figure 10: Opioids – Percentage of patients per year prescribed opioids for patients with and without chronic pain after TKR 11](#_Toc99349765)

[Table 4: Differences in mean prescription costs per patient per year between patients with and without chronic pain after TKR 11](#_Toc99349766)

[Table 5: Yearly prescription costs with bootstrap confidence intervals by chronic pain group 12](#_Toc99349767)

[Figure 11: Sex-stratified mean yearly consultation costs with bootstrap confidence intervals by chronic pain group 13](#_Toc99349768)

[Figure 12: Sex-stratified mean yearly prescription costs with bootstrap confidence intervals by chronic pain group 13](#_Toc99349769)

# Table 1: Staff roles recording in CPRD

| **Included staff roles** | **Excluded staff roles** |
| --- | --- |
| Acupuncturist  Associate  Chiropodist  Chiropractor  Clinical Practitioner Access Role  Commercial Deputising service  Community Medical Officer  Community Nurse  Community Psychiatric Nurse  Consultant  Contact Tracing Nurse  Counsellor  Dietician  GP Registrar  GP Retainer  Health Education Officer  Health Professional Access Role  Health Visitor  Hospital Nurse  Locum  Midwife  Non-commercial local rota of less than 10 GPs  Nurse Access Role  Nurse Manager Access Role  Occupational Therapist  Other Additional Clinical Services  Other Allied Health Professionals  Other Health Care Professional  Other Medical & Dental  Other Nursing & Midwifery  Partner  Physiotherapist  Practice Nurse  Salaried Partner  School Nurse  Senior Partner  Social Worker  Sole Practitioner | Administrator  Assistant  Business Manager  Clinical Coder Access Role  Computer Manager  Data Not Entered  Dispenser  Fund Manager  Interpreter/Link Worker  Maintenance staff  Non-qualified Dispenser  Other Admin & Clerical  Other Healthcare Scientists  Other Professional Scientific & Technical  Other Students  Pharmacist  Phlebotomist  Practice Manager  Radiographer  Receptionist  Secretary |

# Table 2: Inclusion/exclusion criteria for primary care consultations

| **Included consultation types** | **Excluded consultation types** |
| --- | --- |
| Acute visit  Casualty Attendance  Children's Home Visit  Clinic  Co-op Home Visit  Co-op Surgery Consultation  Co-op Telephone advice  Community Clinic  Emergency Consultation  Follow-up/routine visit  Home Visit  Hotel Visit  Minor Injury Service  Night Visit  Night visit , practice  Night visit, Deputising service  Night visit, Local rota  Nursing Home Visit  Out of hours, Non Practice  Out of hours, Practice  Radiology Request  Residential Home Visit  Surgery consultation  Telephone call from a patient  Telephone call to a patient  Telephone Consultation  Third Party Consultation  Twilight Visit  Walk-in Centre | Administration  Community Nursing Note  Community Nursing Report  Data Not Entered  Data Transferred from other system  Day Case Report  Discharge details  ePharmacy message  GOS18 Report  GP to GP communication transaction  Health Authority Entry  Health Visitor Note  Health Visitor Report  Hospital Admission  Hospital Inpatient Report  Initial Post Discharge Review  Laboratory Request  Letter from Outpatients  Mail from patient  Mail to patient  Medicine Management  NHS Direct Report  Non-consultation data  Non-consultation medication data  Other  Radiology Result  Referral Letter  Repeat Issue  Results recording  Social Services Report  Template Entry  Triage |

# Specific drugs included in analysis

Paracetamol tablets, including effervescent and orodispersible, accounted for 93.6% of paracetamol prescriptions, and these together with capsules (6.3%) were included in the analysis. Oral suspensions, powder and suppositories, accounting in total for less than 1% of prescription, were excluded. Prescription records with missing quantities (0.2%) were also excluded.

Antidepressants tablets, including modified-release, accounted for 93.7% of antidepressant prescriptions, and these together with gastro-resistant and modified-release capsules (6.3%) were included in the analysis. Oral suspensions, accounting for less than 1% of antidepressant prescriptions, were excluded. Prescription records with missing quantities (<0.1%) were also excluded.

NSAIDs tablets, including dispersible, gastro-resistant, modified-release, orodispersible, and enteric-coated accounted for 91.0% of NSAIDs prescriptions, and these together with capsules (8.5%) were included in the analysis. Oral suspensions, power, solution for injection, suppositories, and transdermal patches, accounting in total for less than 1% of all NSAIDs prescriptions, were excluded. Prescription records with missing quantities (1.6%) were also excluded.

Opioid capsules, including modified-release, accounted for 61.2% of opioids prescriptions, and these together with tablets, including modified-release, prolonged-release, orodispersible, soluble, and sublingual (22.6%) and patches (11.4%) were included in the analysis. Injections, oral solutions, power, and solution for injection accounting in total for 4.8% of all opioids prescriptions were excluded given the lack of sufficient information (quantity, dose, etc) to cost them. Prescription records with missing quantities (3.0%) were also excluded.


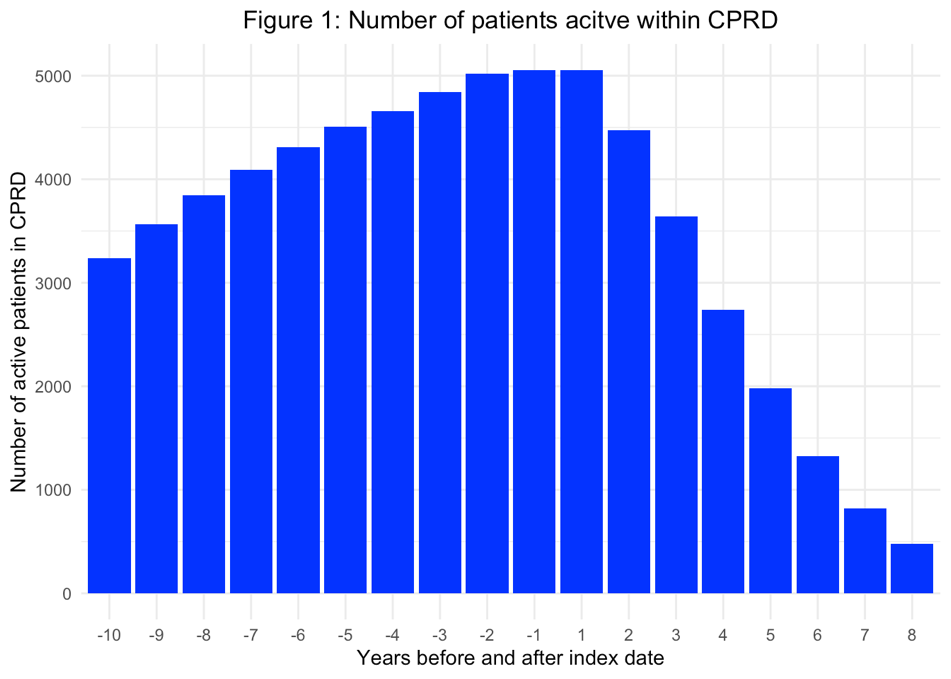


# Figure 1: Number of patients active within CPRD by year before and after primary TKR


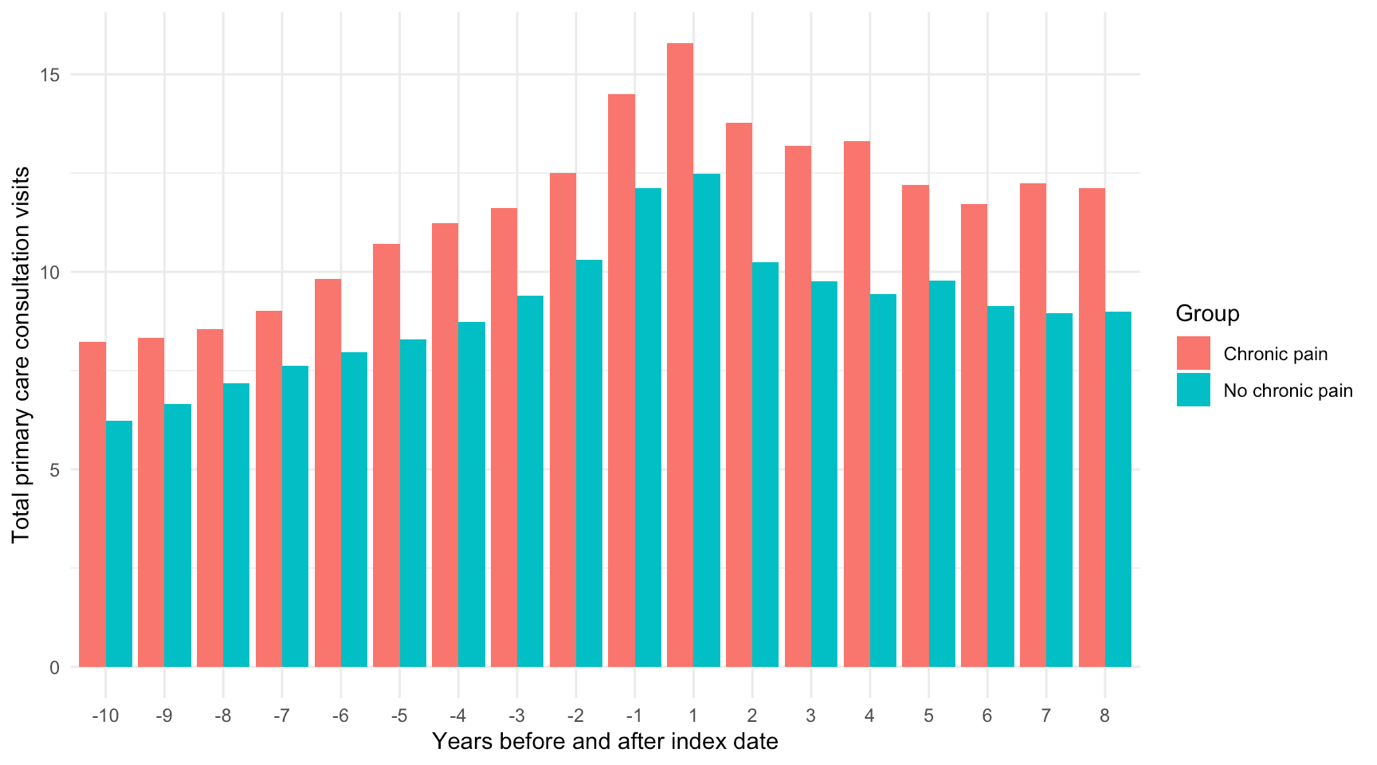


# Figure 2: Mean number of primary care consultations by year for patients with and without chronic pain after TKR


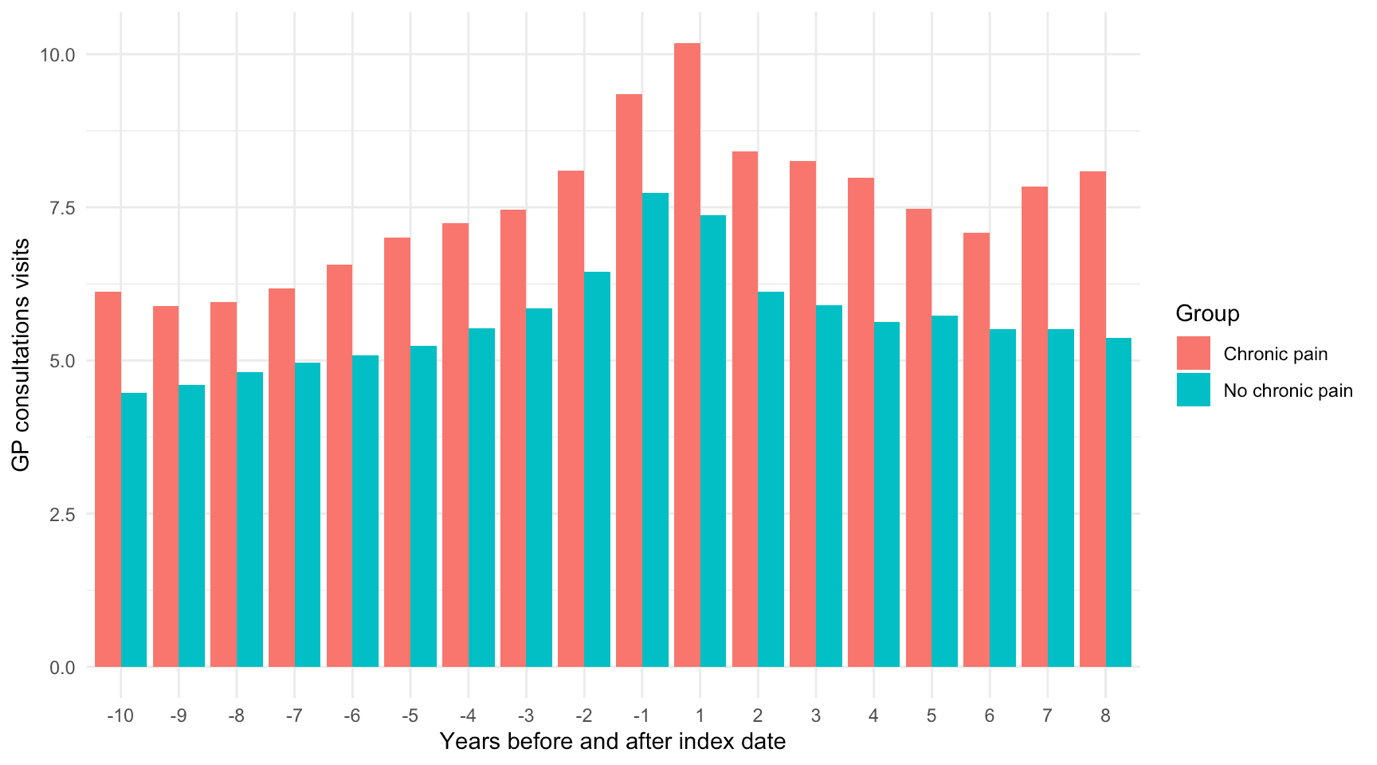
Figure 3: Mean number of GP consultations by year for patients with and without chronic pain after TKR


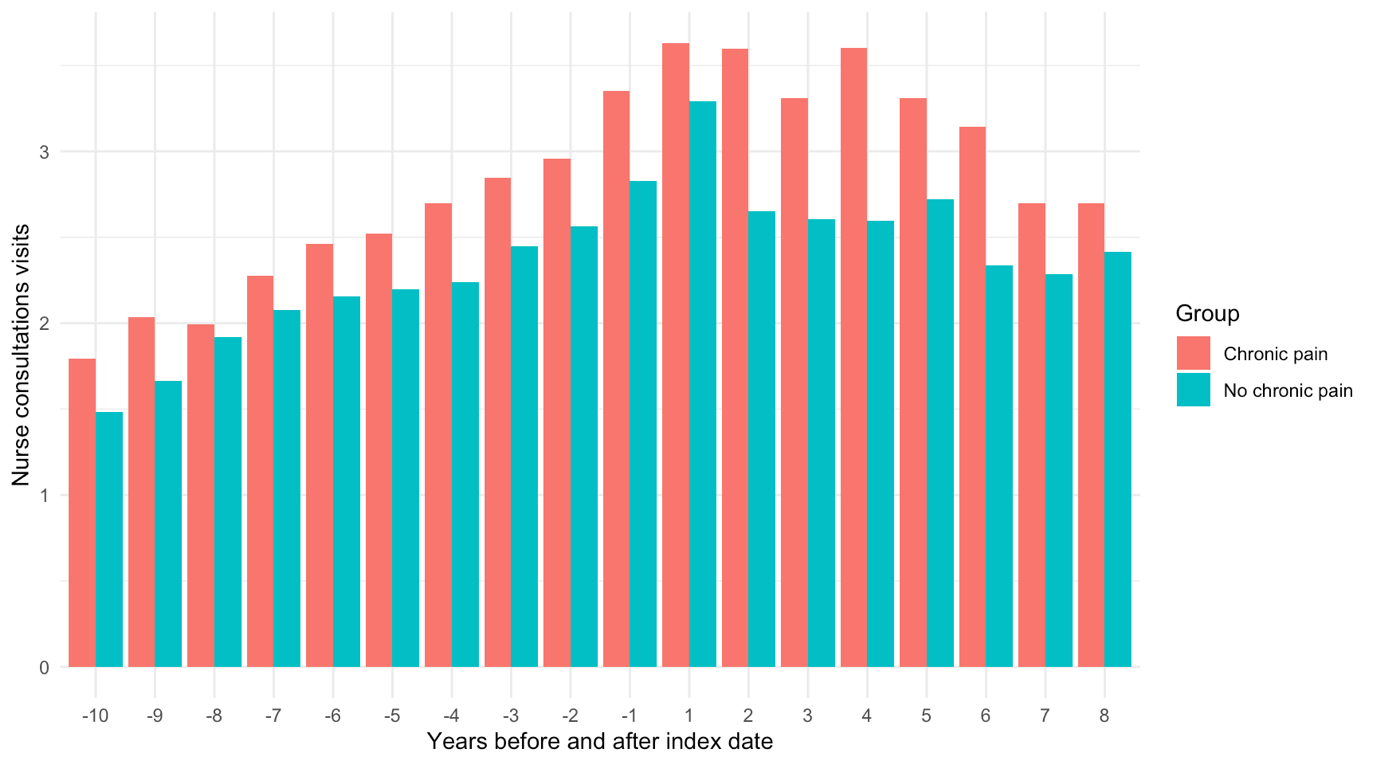


# Figure 4: Mean number of nurse consultations by year for patients with and without chronic pain after TKR


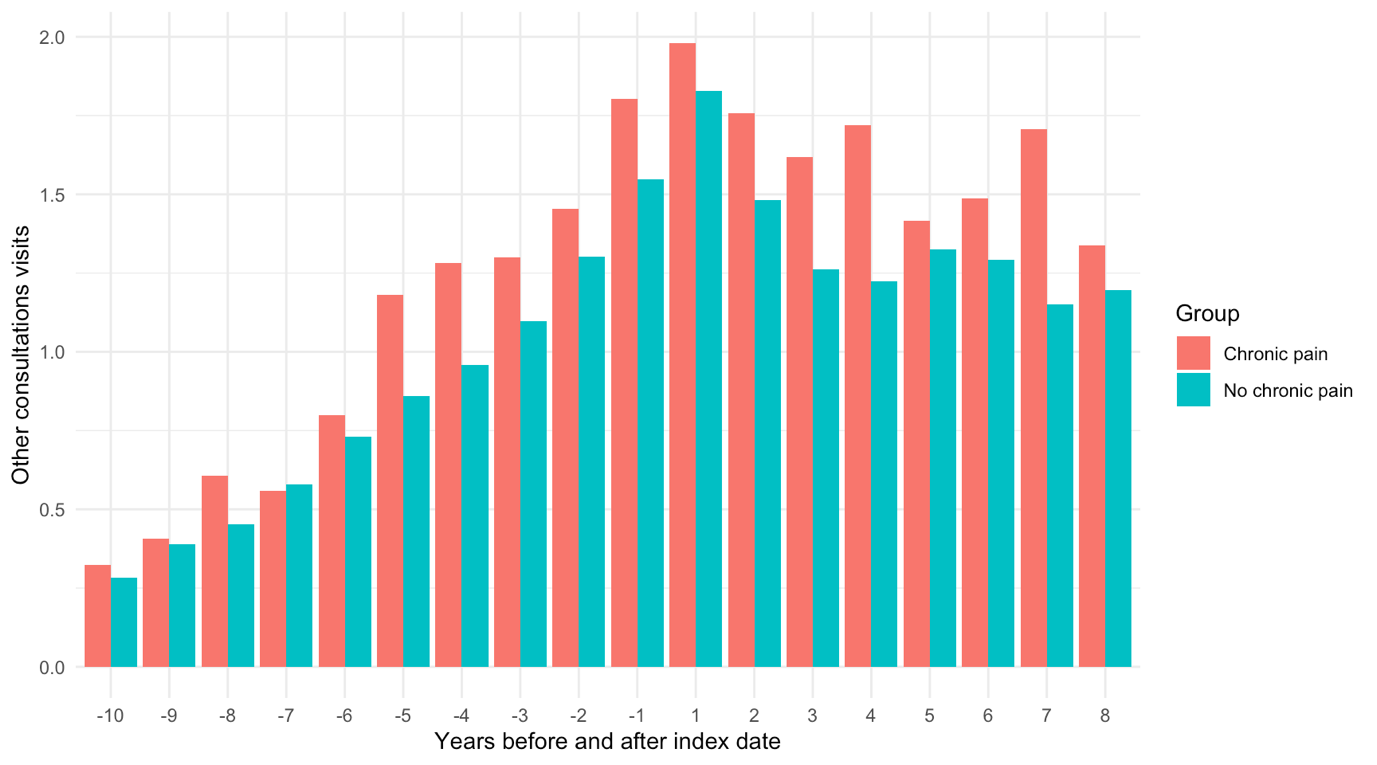


# Figure 5: Mean number of other healthcare professional consultations by year for patients with and without chronic pain after TKR

# **Table 3: Mean number of consultations for the different types of healthcare professionals by year** for patients with and without chronic pain after TKR

|  | **With chronic pain** | | | | **Without chronic pain** | | | |
| --- | --- | --- | --- | --- | --- | --- | --- | --- |
| **Year** | **GPs** | **Nurses** | **Other** | **Total** | **GPs** | **Nurses** | **Other** | **Total** |
| -10 | 6.12 | 1.79 | 0.32 | 8.24 | 4.47 | 1.48 | 0.28 | 6.23 |
| -9 | 5.89 | 2.03 | 0.41 | 8.33 | 4.61 | 1.67 | 0.39 | 6.66 |
| -8 | 5.95 | 1.99 | 0.61 | 8.55 | 4.81 | 1.92 | 0.45 | 7.18 |
| -7 | 6.17 | 2.28 | 0.56 | 9.01 | 4.97 | 2.08 | 0.58 | 7.63 |
| -6 | 6.57 | 2.46 | 0.80 | 9.83 | 5.09 | 2.15 | 0.73 | 7.97 |
| -5 | 7.00 | 2.52 | 1.18 | 10.70 | 5.24 | 2.20 | 0.86 | 8.29 |
| -4 | 7.25 | 2.70 | 1.28 | 11.23 | 5.53 | 2.24 | 0.96 | 8.73 |
| -3 | 7.46 | 2.85 | 1.30 | 11.61 | 5.84 | 2.45 | 1.10 | 9.39 |
| -2 | 8.10 | 2.96 | 1.45 | 12.51 | 6.45 | 2.56 | 1.30 | 10.31 |
| -1 | 9.35 | 3.35 | 1.80 | 14.50 | 7.74 | 2.83 | 1.55 | 12.11 |
| 1 | 10.18 | 3.63 | 1.98 | 15.79 | 7.37 | 3.29 | 1.83 | 12.48 |
| 2 | 8.41 | 3.60 | 1.76 | 13.76 | 6.12 | 2.65 | 1.48 | 10.25 |
| 3 | 8.26 | 3.31 | 1.62 | 13.19 | 5.90 | 2.60 | 1.26 | 9.76 |
| 4 | 7.98 | 3.60 | 1.72 | 13.30 | 5.63 | 2.60 | 1.22 | 9.45 |
| 5 | 7.47 | 3.31 | 1.42 | 12.20 | 5.73 | 2.72 | 1.32 | 9.77 |
| 6 | 7.08 | 3.14 | 1.49 | 11.71 | 5.51 | 2.34 | 1.29 | 9.14 |
| 7 | 7.84 | 2.70 | 1.71 | 12.25 | 5.51 | 2.28 | 1.15 | 8.95 |
| 8 | 8.08 | 2.70 | 1.34 | 12.12 | 5.37 | 2.42 | 1.20 | 8.98 |


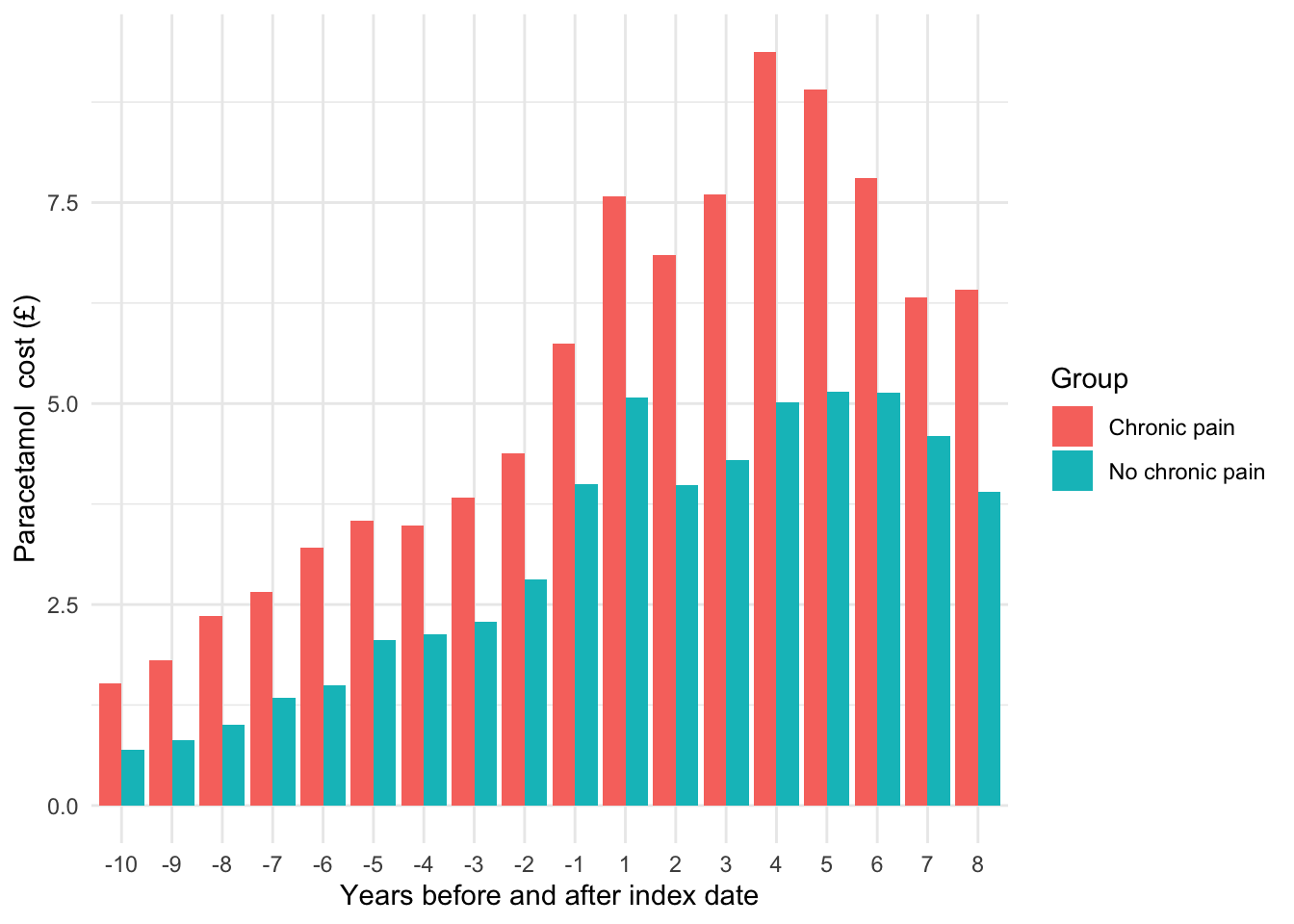


# **Figure 6: Paracetamol - Mean cost per patient per year for** patients with and without chronic pain after TKR


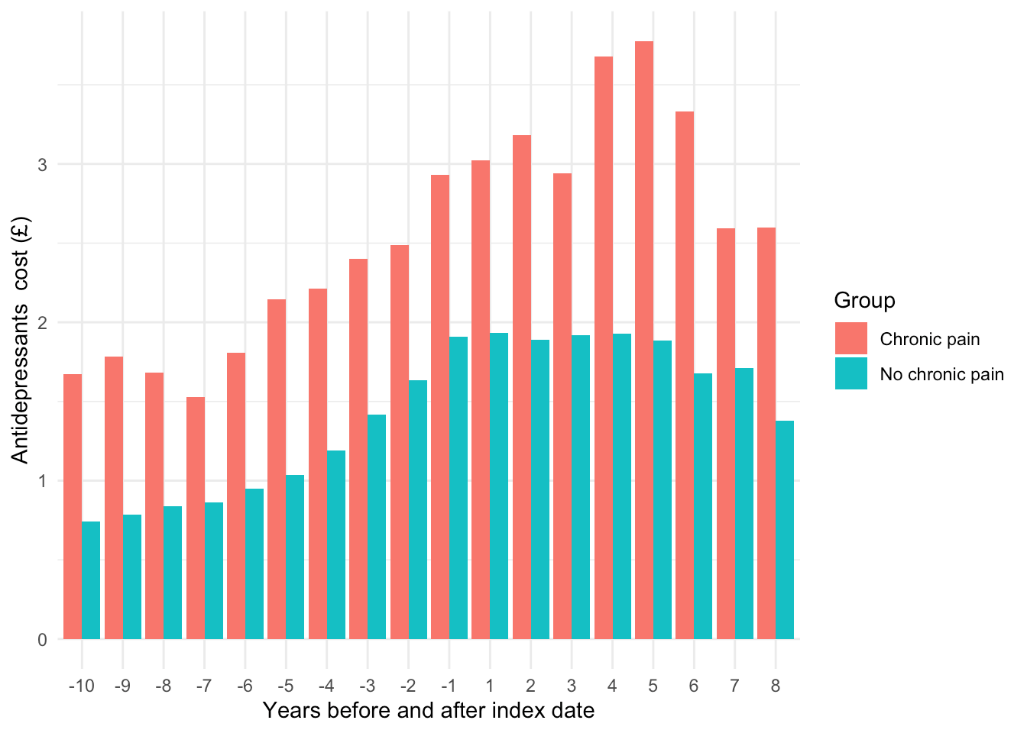


# Figure 7: Antidepressants - Mean cost per patient per year for patients with and without chronic pain after TKR


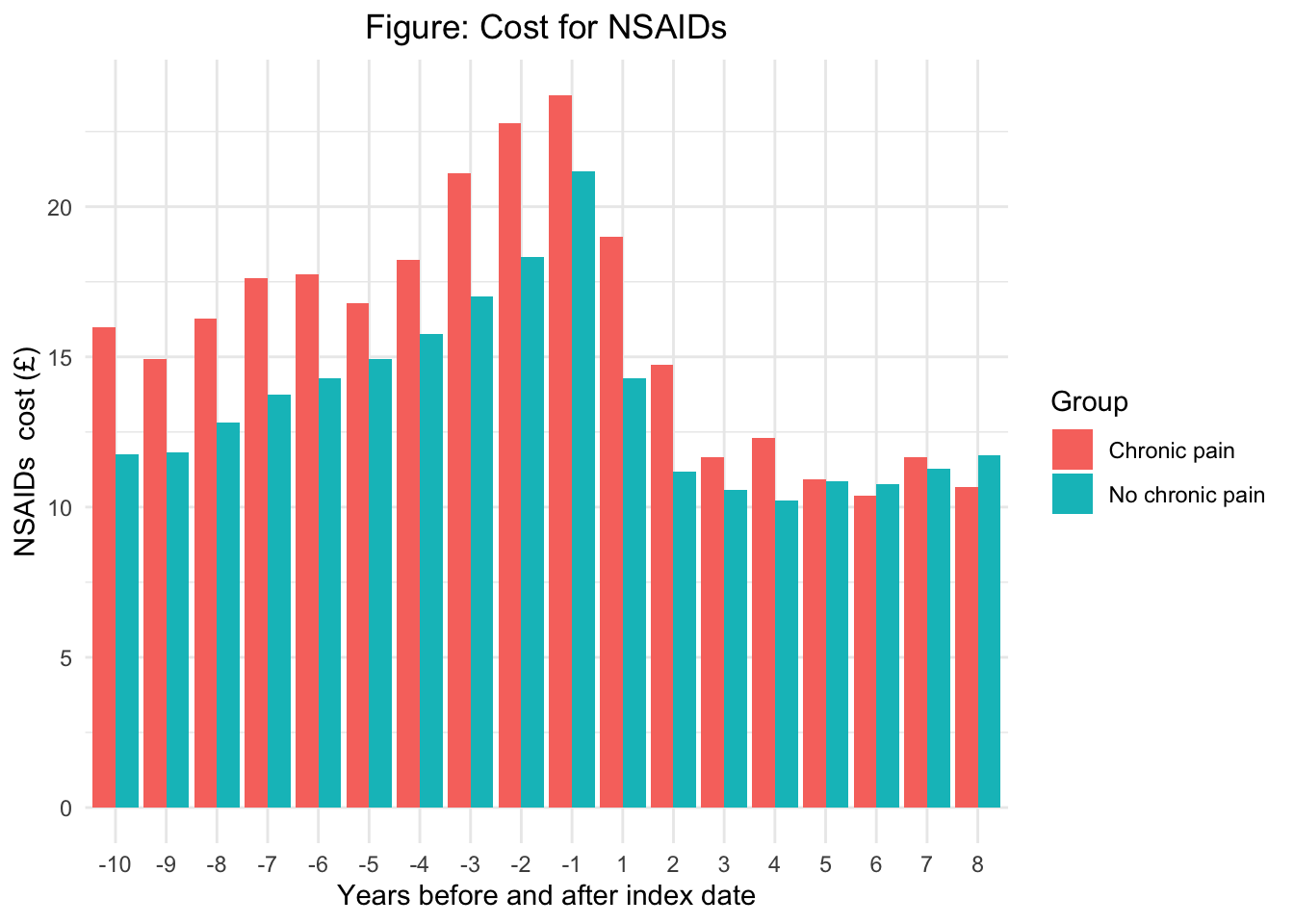


# Figure 8: NSAIDs - Mean cost per patient per year for patients with and without chronic pain after TKR


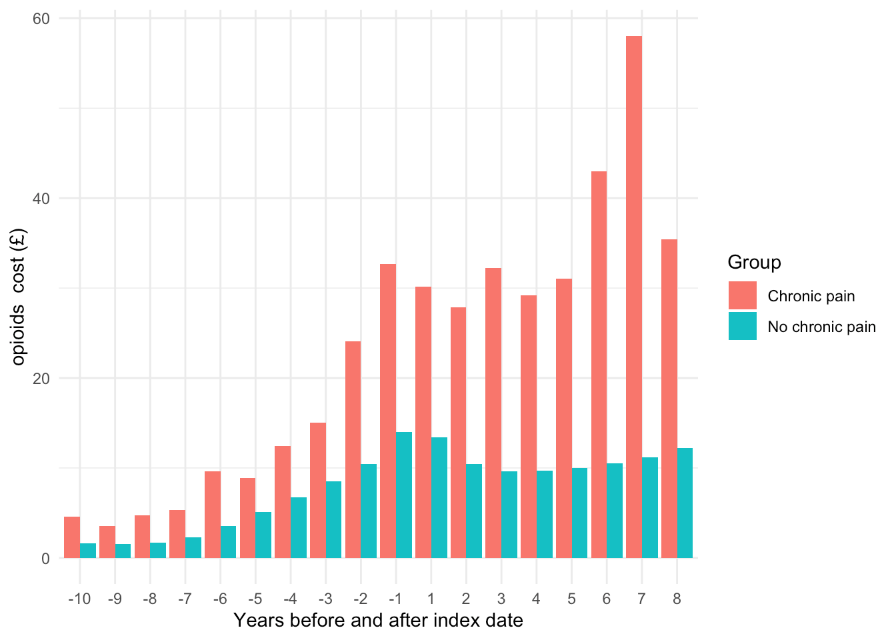


# Figure 9: Opioids - Mean cost per patient per year for patients with and without chronic pain after TKR


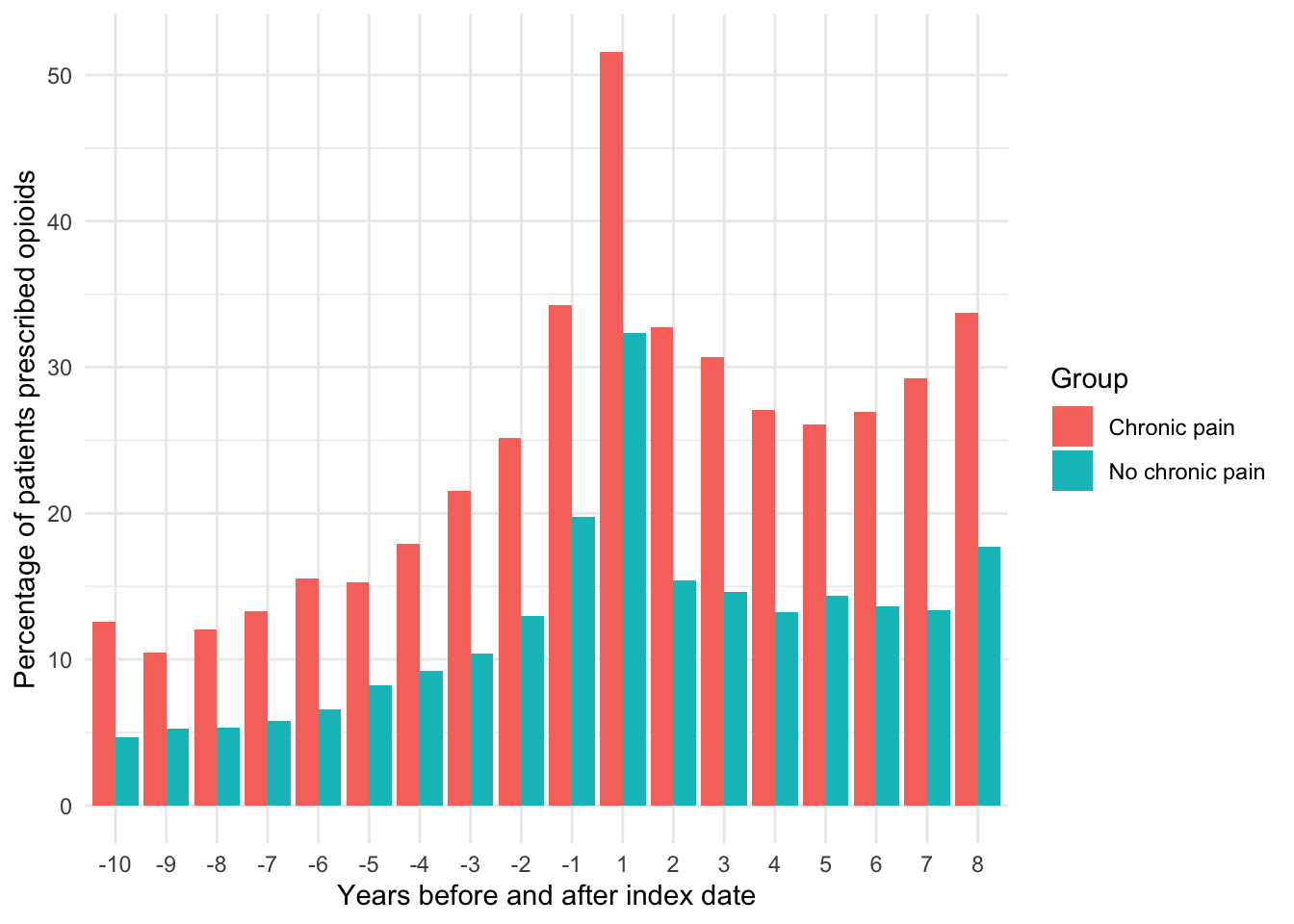


# Figure 10: Opioids – Percentage of patients per year prescribed opioids for patients with and without chronic pain after TKR

# Table 4: Differences in mean prescription costs per patient per year between patients with and without chronic pain after TKR

| **Year** | **Paracetamol** | **Antidepressants** | **NSAIDs** | **Opioids** | **Total** |
| --- | --- | --- | --- | --- | --- |
| -10 | £0.83 | £0.93 | £0.93 | £5.06 | £7.75 |
| -9 | £1.00 | £1.00 | £1.00 | £3.94 | £6.94 |
| -8 | £1.35 | £0.84 | £0.84 | £5.72 | £8.75 |
| -7 | £1.32 | £0.67 | £0.67 | £6.34 | £9.00 |
| -6 | £1.71 | £0.86 | £0.86 | £8.22 | £11.65 |
| -5 | £1.48 | £1.10 | £1.10 | £4.30 | £7.98 |
| -4 | £1.35 | £1.02 | £1.02 | £6.99 | £10.38 |
| -3 | £1.55 | £0.98 | £0.98 | £9.55 | £13.06 |
| -2 | £1.57 | £0.86 | £0.86 | £17.37 | £20.66 |
| -1 | £1.75 | £1.02 | £1.02 | £20.16 | £23.95 |
| 1 | £2.50 | £1.09 | £1.09 | £20.39 | £25.07 |
| 2 | £2.87 | £1.29 | £1.29 | £19.70 | £25.15 |
| 3 | £3.30 | £1.02 | £1.02 | £22.64 | £27.98 |
| 4 | £4.37 | £1.75 | £1.75 | £19.82 | £27.69 |
| 5 | £3.77 | £1.89 | £1.89 | £19.22 | £26.77 |
| 6 | £2.66 | £1.65 | £1.65 | £30.43 | £36.39 |
| 7 | £1.72 | £0.89 | £0.89 | £46.45 | £49.95 |
| 8 | £2.50 | £1.22 | £1.22 | £20.99 | £25.93 |

# Table 5: Yearly prescription costs with bootstrap confidence intervals by chronic pain group

|  | **Chronic pain** | | **Non chronic pain** | |
| --- | --- | --- | --- | --- |
| **Year** | **Mean** | **Confidence interval** | **Mean** | **Confidence interval** |
| -10 | 20.66 | 18.92 - 26.46 | 12.91 | 12.34 - 14.79 |
| -9 | 20.14 | 18.78 - 24.7 | 13.20 | 12.77 - 14.67 |
| -8 | 23.54 | 21.96 - 28.85 | 14.79 | 14.34 - 16.34 |
| -7 | 26.02 | 24.44 - 31.64 | 17.02 | 16.51 - 18.77 |
| -6 | 31.07 | 29.09 – 38.00 | 19.42 | 18.69 - 21.88 |
| -5 | 30.51 | 28.86 - 36.39 | 22.53 | 21.69 - 25.12 |
| -4 | 35.65 | 33.48 - 43.21 | 25.27 | 24.41 - 28.02 |
| -3 | 41.97 | 39.82 - 50.22 | 28.91 | 27.89 - 32.17 |
| -2 | 53.80 | 49.81 - 67.59 | 33.14 | 32.07 - 36.55 |
| -1 | 65.05 | 60.49 - 79.5 | 41.10 | 39.98 - 44.79 |
| 1 | 59.76 | 57.18 - 69.57 | 34.69 | 33.76 - 37.54 |
| 2 | 52.62 | 49.92 - 63.09 | 27.47 | 26.58 - 30.19 |
| 3 | 54.40 | 49.96 - 69.75 | 26.42 | 25.50 - 29.42 |
| 4 | 54.53 | 50.13 - 70.24 | 26.84 | 25.74 - 30.28 |
| 5 | 54.62 | 49.74 - 71.84 | 27.85 | 26.73 - 31.72 |
| 6 | 64.47 | 55.75 - 93.16 | 28.08 | 26.56 - 33.51 |
| 7 | 78.70 | 63.64 - 127.57 | 28.75 | 26.70 - 36.52 |
| 8 | 55.11 | 45.92 - 82.98 | 29.18 | 26.47 - 37.29 |


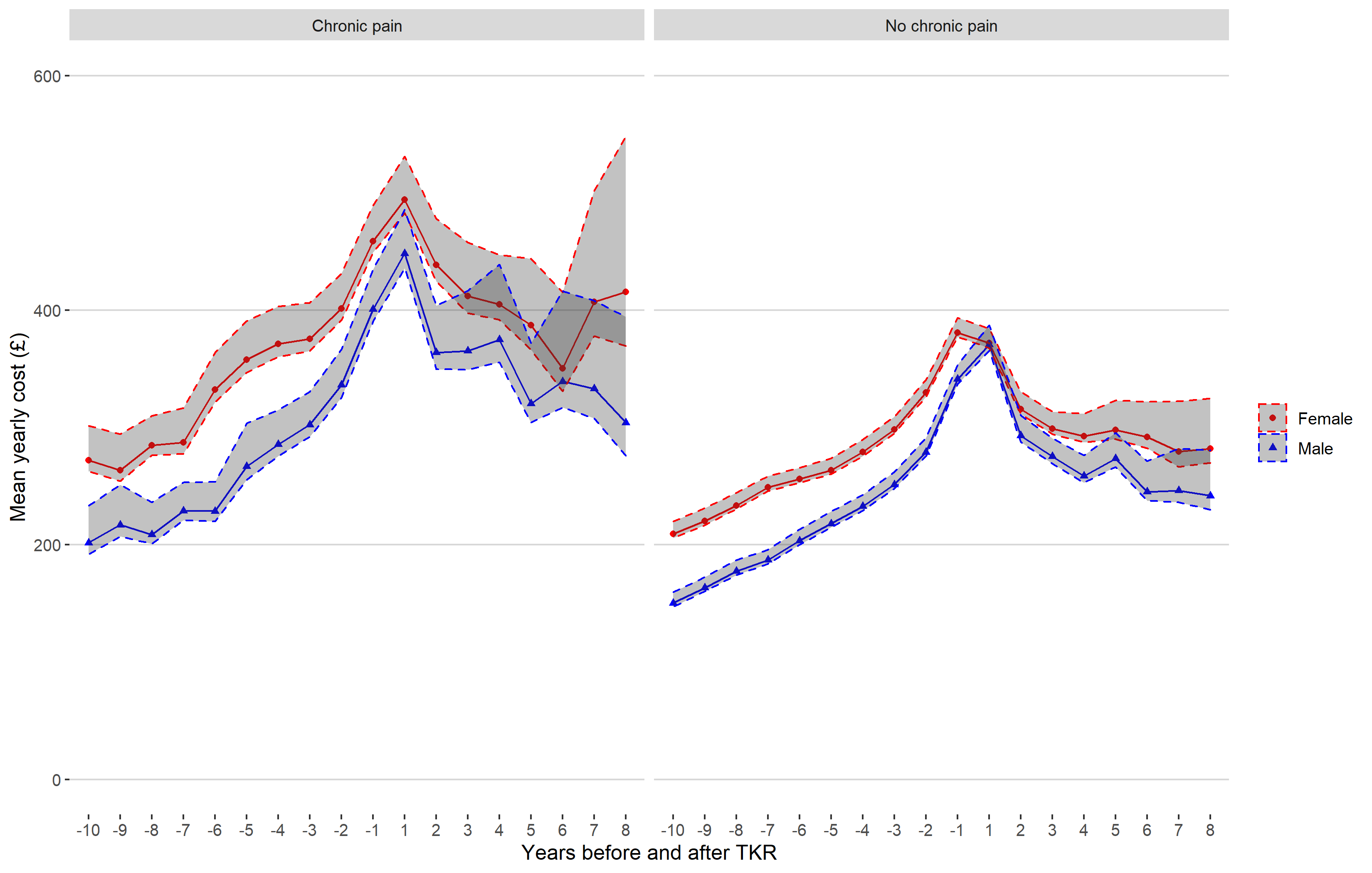


# Figure 11: Sex-stratified mean yearly consultation costs with bootstrap confidence intervals by chronic pain group


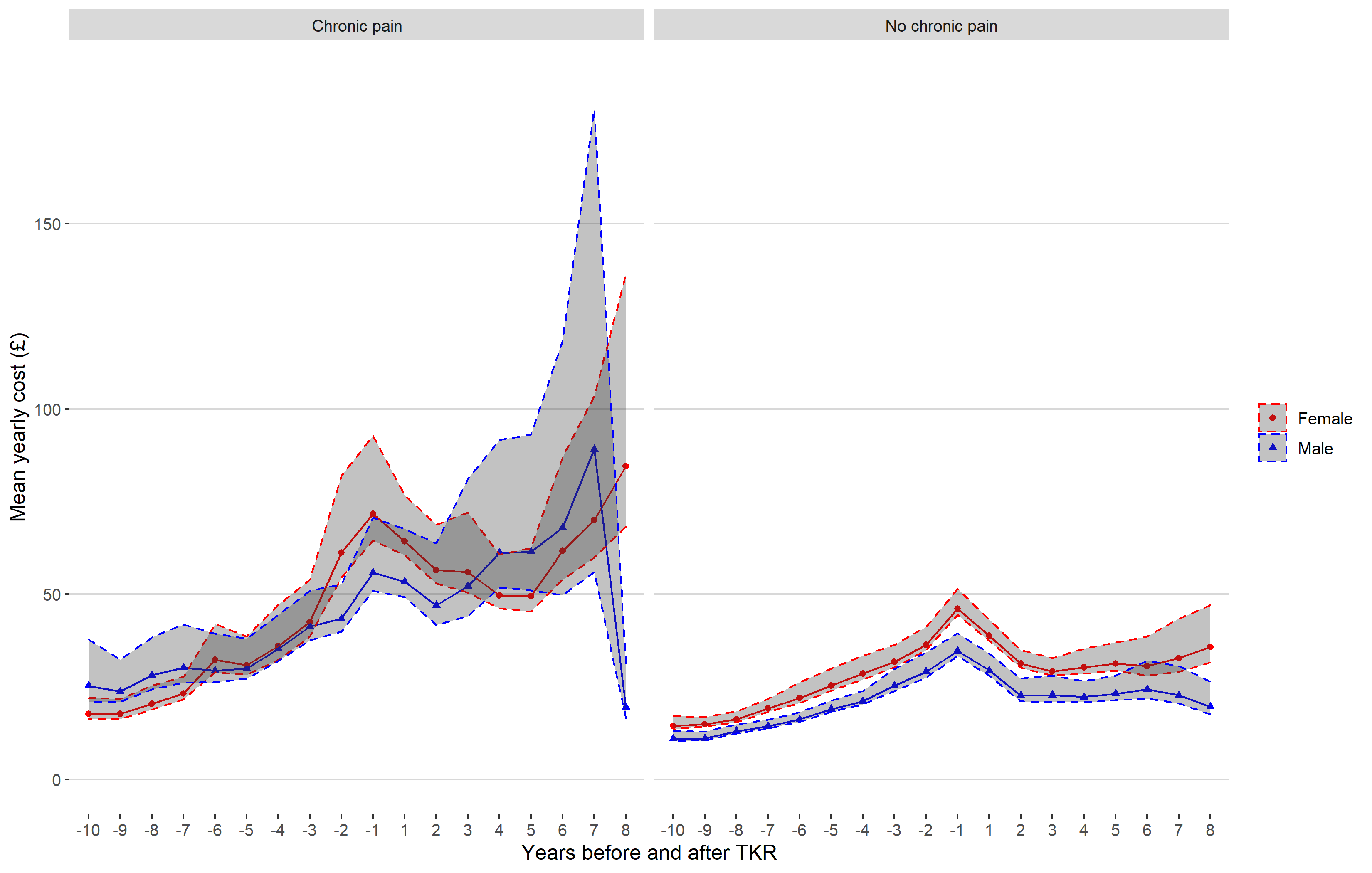


# Figure 12: Sex-stratified mean yearly prescription costs with bootstrap confidence intervals by chronic pain group
